# The Senses

**Published:** 1889-07

**Authors:** 


THE SENSES: FIVE OR SEVEN?—We are in receipt of an exceedingly interesting paper with the above caption, being an abstract of a paper read before the New York Academy of Anthropology, March, 1889, by Dr. Wm. M. McLaury of New York.

The many strange psychical phenomena of modern times have given rise to new theories upon subjects pertaining to to the brain and nervous system. In the paper above referred to Dr. McLaury describes what has been regarded as the seventh or magnetic sense—under which may perhaps be included the faculty which enables birds and animals to find their homes from a distance. Also the effects of sympathy by which a person sometimes seems to realize in his own body something of the sufferings of an injury to another; even though that injury may occur at a distant point. And we suppose that certain premonitions of approaching events and trance conditions would be attributed to the magnetic sense. Our space forbids further comments upon the points in the paper referred to, which are certainly suggestive and interesting.

